# The impact of oocytes containing smooth endoplasmic reticulum aggregates on assisted reproductive outcomes: a cohort study

**DOI:** 10.1186/s12884-022-05141-9

**Published:** 2022-11-14

**Authors:** Tingfeng Fang, Wenchang Yu, Songbang Ou, Jinyu Lu, Ruiqi Li, Mingpeng Zhao, Yiu Leung Chan, Wenjun Wang

**Affiliations:** 1 Guangdong Provincial Key Laboratory of Malignant Tumor Epigenetics and Gene Regulation, Sun Yat-Sen Memorial Hospital, Sun Yat-Sen University, Guangzhou, China; 2 Department of Obstetrics & Gynecology, Sun Yat-Sen Memorial Hospital, Sun Yat-Sen University, Guangzhou, China; 3 Department of Reproductive Medicine, Department of Obstetrics and Gynaecology, Guangdong Provincial People’s Hospital, Guangdong Academy of Medical Sciences, Guangzhou, China; 4 Assisted Reproductive Technology Unit, Department of Obstetrics and Gynecology, Faculty of Medicine, Chinese University of Hong Kong, Hongkong, China

**Keywords:** Smooth endoplasmic reticulum aggregates (SERa), Oocyte, Dysmorphism, Reproductive outcomes, IVF, ICSI

## Abstract

**Background:**

The impact of smooth endoplasmic reticulum aggregates (SERa) on assisted reproductive technology (ART) outcomes was still controversial. Our objective is to investigate the impact of the presence of SERa on intracytoplasmic sperm injection (ICSI) outcomes.

**Methods:**

This was a retrospective cohort study. A total of 1,090 fresh ICSI cycles from 944 patients between January 2016 and June 2020 were included. Outcomes from clinical, embryological and neonatal aspects were compared between SERa + and SERa- cycles as well as between SERa + and SERa- oocytes.

**Results:**

The total gonadotropin (Gn) dose, number of oocytes retrieved, serum estradiol concentration and number of the available embryo were significantly higher in SERa + cycles than in SERa- cycles (*P* < 0.05). Comparable two pronuclei (2PN) fertilization rate and poly-pronucleus zygote rate were shown in SERa + and SERa- cycles (*P* > 0.05), but which were higher in SERa + oocytes than in SERa- oocytes (*P* < 0.05). No statistical difference in blastocyst formation rate was found in SERa + and SERa- cycles as well as in SERa + and SERa- oocytes (*P* > 0.05). Good-quality embryo rate was statistically higher in SERa- cycles than in SERa + cycles (*P* < 0.05), but the difference was comparable between SERa + and SERa- oocytes (*P* > 0.05). No statistical difference in clinical pregnancy rate, spontaneous abortion rate, live birth rate and premature delivery rate were found in SERa + and SERa- cycles as well as in SERa + and SERa- oocytes (*P* > 0.05). The implantation rate was comparable in SERa + and SERa- cycles (*P* > 0.05), but it is higher in the group of only SERa- embryo transfer when compared with the group of mixed SERa + and SERa- embryo transfer (*P* < 0.05). 159 newborns in SERa + cycles and 140 newborns in SERa- cycles were followed up. Comparable newborn malformation rate was observed between SERa + and SERa- cycles and oocytes (*P* > 0.05). Logistic regression analysis revealed number of oocytes and total dose of Gn were risk factors for SERa occurrence (aOR = 1.05 and 1.55, *P* < 0.001).

**Conclusion:**

Oocyte's SERa is correlated with a number of oocytes retrieved and higher Gn dose, but it does not affect pregnancy outcomes and increase newborn malformation rate.

**Supplementary Information:**

The online version contains supplementary material available at 10.1186/s12884-022-05141-9.

## Introduction

Smooth endoplasmic reticulum aggregates (SERa) in the oocyte are a type of cytoplasmic dysmorphism, showing smooth vacuoles that appear as central round flat discs in the cytoplasm [1]. The release of calcium in SER plays a crucial role in oocyte maturation and fertilization as well as in early embryo development [2, 3]. The presence of SERa in the oocyte is correlated with dysregulation of calcium signaling and may affect oocyte fertilization in clinical in vitro fertilization (IVF) practices [4].

The presence of SERa in assisted reproductive technology (ART) cycles were associated with lower chances of successful pregnancy [5], neonatal imprinting disorders [5] and a higher incidence of perinatal complications [6]. Multiple fetal anomalies were found in the pregnancies of the woman with SERa + oocytes [7]. In view of these results, in 2011 the Istanbul Consensus advised that oocytes with SERa should not be injected/inseminated and transferred [8]. Several studies also reported that the presence of SERa in oocytes was also associated with cleavage failure [9], lower fertilization rate [10], lower implantation rates [11] and blastocyst formation rates [12]. However, other studies have revealed that SERa + cycles and oocytes are not completely negatively correlated with embryological, clinical and neonatal outcomes, and normal healthy babies can be born from embryos derived from from SERa + oocytes [13–16]. The exclusion of SERa + oocytes from intracytoplasmic sperm injection (ICSI) cycles can cause an increased frequency of transfer cancellation [17]. In 2017, the Vienna Consensus reconsidered the recommendation of the Istanbul Consensus and advised on a case-by-case basis [18]. Since there was no effective method for SERa management, it was suggested that both SERa- and SERa + oocytes can be fertilized and that embryos derived from SERa- oocytes should be prioritized [19].

So far, most studies about the impact of SERa on ART outcomes were small sample sizes. A recent study with 331 neonates’ follow-up was conducted to analyse the impact of SERa on ART outcomes [20], however, the newborns in the study were all from SERa- oocytes. Due to medical ethics, randomized controlled studies are almost impossible to implement. In evidence-based medicine, we need a larger sample of retrospective analysis including newborns from SERa + and SERa- oocytes to provide more insight into the safety of SERa + oocytes. The current study conducted a retrospective analysis from 1090 fresh ICSI cycles with 299 neonates from SERa + or SERa- cycles (including the babies exclusively derived from SERa + oocytes) follow-up to investigate the impact of SERa on ART outcomes.

## Materials and methods

### Study design and patients

**Fig. 1:**
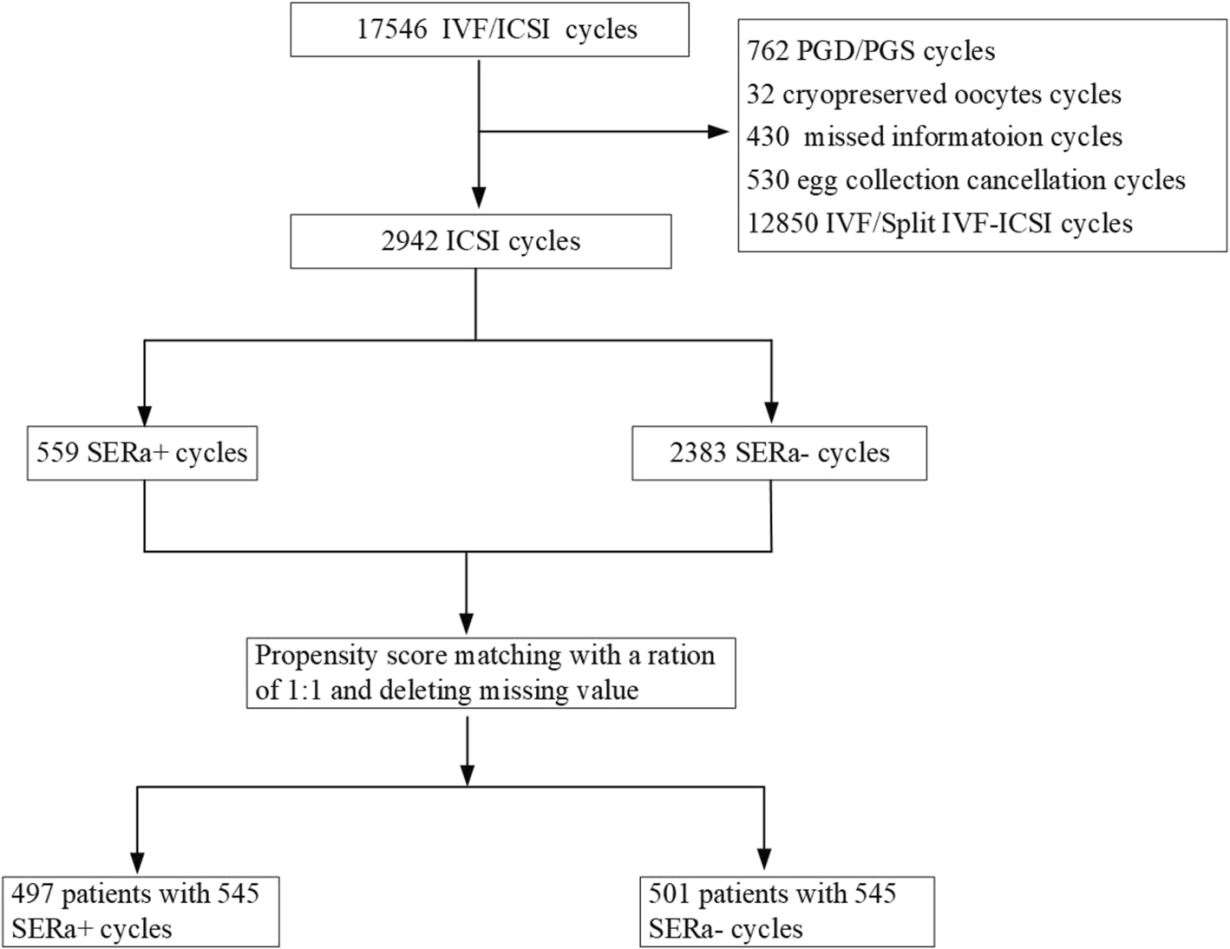
Patient selection flowchart

This was a retrospective cohort study of fresh ICSI cycles from women who were treated at the Reproduction Center of Sun Yat-sen Memorial Hospital, Sun Yat-sen University between January 2016 and December 2020. Patients undergoing IVF and split IVF-ICSI Cycles, egg collection cancellation, preimplantation genetic diagnosis (PGD)/preimplantation genetic screening (PGS) and records with misinformation were excluded. The sample selection details are shown in Fig. 1. In total, 998 patients with 1090 ICSI cycles were included in this study, the newborn outcomes were followed up by a trained nurse through a telephone call.

### Ovarian stimulation, insemination, and the ICSI procedure

The details for ovarian stimulation and egg collection in our center have been described in our previous study [21, 22]. Gonadotropin-releasing hormone agonist (GnRHa, Decapeptyl; Ferring GmbH) protocol was defined that ovarian stimulation started with daily injection of recombinant follicle-stimulating hormone (rFSH; Gonal-F, Merck Serono) including or not GnRHa for pituitary desensitization in luteal phase. GnRH antagonist (Cetrotide, Merck Serono) protocol was defined that GnRH antagonist was uesed in ovarian stimulation and GnRHa 0.2 mg + HCG 2000 IU was injected when three follicles were ≥ 16 mm, two follicles were ≥ 17 mm, or one follicle was ≥ 18 mm in diameter and serum E2 levels reached or exceeded the level that corresponded to the size and number of follicles. Other protocols included minimal stimulation protocol with rFSH or human menopausal gonadotrophin (HMG; Menopur; Ferring Pharmaceuticals, Tokyo, Japan), clomiphene citrate protcol (Serophene; Merck Serono), letrozole protocol (Novartis Pharma Schweiz AG, Switzerland) and natural cycle. Routine ICSI was conducted after egg collection for 3–4 h. The appearance of two pronuclei (2PN), at 16–18 h post-insemination was considered normal fertilization. Two to three embryos either from day 3 or day 5 were selected for transfer depending on the quality of the embryos. Patients with positive human chorionic gonadotropin (hCG) values in the serum or morning urine on the 14th day after embryo transfer were preliminarily diagnosed as having a biochemical pregnancy, and clinical pregnancy was diagnosed when a pregnancy sac and fetal heartbeat were confirmed by ultrasound examination during the 5th week after transplantation.

### Evaluation of SERa

In the ICSI procedure, the denudation was performed 3–4 h after oocyte collection. The presence of SERa was recorded during the ICSI procedure. The appearance of SERa is similar to vacuoles in the oocyte’s cytoplasm, which can be easily distinguished from a vacuole since it is not fluid-filled and not separated from the rest of the cytoplasm by a membrane [14].

### Statistical analysis

SPSS software (version 20.0, IBM Corporation, USA) was used to analyze the data. Data with a normal distribution and continuous variables were recorded as the mean ± standard deviation and data without normal distributions were recorded as the median (interquartile range). Frequency data were recorded as % (n/N).

We used the female age, body mass index (BMI) and basal follicle-stimulating hormone (bFSH) to match women from SERa- cycles through propensity score matching by the R program (version 3.4.1). The sample size were 545 and 545 in the SERa + and SERa- groups with a ratio of 1:1, the power was calculated to be 80%, details information are shown in the following sample size calculation.

Since the data of multiple cycles per patient may be non-independent, we first used a 2-level (i.e., levels 1 and 2 were cycle and patient respectively) binary logistic regression zero model of SERa to check whether a clustering in level 2 was exist using MLwiN 2.10 software (The Center for Multilevel Modeling, UK). The results indicated the dataset was not clustered at level 2 (Table 1). Thus, continuous data were compared between groups via t-tests or one-way ANOVA and the qualitative data were compared between groups with the chi-square (χ^2^) test or Mann–Whitney test. Univariate analysis and multivariate logistic regression were performed to evaluate the SERa risk factors. Receiver operating characteristic (ROC) curves were used to evaluate the predictive value of the risk factors of SERa. The test level was set at α = 0.05, and statistical significance was set at *P* < 0.05.

**Table 1:** 2-level logistic zero model of SERa

Variable	Estimated value	SE	*P*
Fixed part (intercept)	-0.000	0.063	1.000
Random part			
${\sigma }_{{\mu }_{0}}^{2}$	0.228	0.164	0.164
$\delta$	1	0.000	-

*SE* stand error;${{\varvec{\sigma}}}_{{{\varvec{\mu}}}_{0}}^{2}$:level 2 variance

$$\delta$$:1evel 1 scale parameter

### Sample size calculation

The formula for sample size estimation:

$${\varvec{n}}=\frac{{\left[{{\varvec{Z}}}_{\propto /2}\sqrt{{\varvec{p}}(1-{\varvec{p}})(1+{\varvec{c}})/{\varvec{c}}}+{{\varvec{Z}}}_{{\varvec{\beta}}}\sqrt{{{\varvec{p}}}_{1}\left(1-{{\varvec{p}}}_{1}\right)+{{\varvec{p}}}_{2}(1-{{\varvec{p}}}_{2}/{\varvec{c}}}\right]}^{2}}{{({{\varvec{p}}}_{1}-{{\varvec{p}}}_{2})}^{2}}$$

According to a previous systematic review [19] that the major malformation rate in newborns was 6.0% in SERa + cycles and 2.1% in SERa- cycles and using the rate of the major malformation in newborns as the primary outcome measurement, based on the formula for two-group design sample size estimation and with an accuracy index α = 0.05 and power = 80% in a bilateral variability test, if SERa + cycles and SERa- cycles were matched 1:1 in this study, the total sample size would be 500 subjects in SERa + cycles and 400 subjects in SERa- cycles. Thus, our sample size achieved this requirement with 545 in the SERa + cycles and 545 in the SERa- cycles.

## Result

### Clinical characteristics, embryo development and pregnancy outcomes

There were 2,942 ICSI cycles in our center between January 2016 and December 2020. Among them, 559 cycles showed at least one SERa + oocytes (19.00%). In our last included 545 SERa + cycles, there were 1,715 SERa + oocytes (25.57%) and 4,991 SERa- oocytes (74.43%). The total Gn dose, number of oocytes retrieved, serum estradiol concentration and number of the available embryo were significantly higher in SERa + cycles than in SERa- cycles. There was a significant difference in the stimulation protocol between the two groups, the further pairwise comparison was shown the proportion of other protocols was higher in the SERa + cycles than in the SERa- cycles (*P* < 0.05, Table 2), no significant difference in the the proportion of GnRHa agonist and antagonist protocol between the two groups (*P* > 0.05, Table 2).

**Table 2:** Clinical characteristics and embryo development of all patients in SERa + and SERa– cycles

Characteristic	SERa + cycles (n = 545)	SERa- cycles (n = 545)	*t/χ*^*2*^*/z*	*P-*value
Age, y	33.90 ± 5.14	33.97 ± 5.63	-0.208	0.835
BMI, kg/m^2^	22.10 ± 3.11	22.13 ± 3.12	-0.152	0.879
Years infertile, y	5.62 ± 3.92	5.72 ± 4.23	-0.405	0.686
Type of infertility			0.381	0.537
Primary infertility	49.20 (320/545)	50.80 (330/545)		
Secondary infertility	51.10 (225/545)	48.90 (215/545)		
Basal FSH, IU/L	8.15 ± 3.53	8.17 ± 3.50		0.923
Protocol, %			11.340	0.003
Agonist	58.00 (316/545)	60.60(330/545)		
Antagonist	27.20 (148/545)	31.00 (169/545)		
Others	14.90 (84/545)	8.40 (46/545)		
Total dose of Gn, IU	2199.13 ± 928.94	2071.28 ± 880.37	2.324	0.020
Initial dose of Gn, IU	192.82 ± 67.01	188.96 ± 63.86	0.970	0.332
Duration of Gn, days	10.68 ± 2.60	10.35 ± 2.94	1.956	0.051
No. of oocytes retrieved, n	12.30 ± 7.19	9.89 ± 7.14	0.873	< 0.001
No. of SERa- oocytes, n	9.16 ± 7.12	9.89 ± 7.14	-1.690	0.091
Serum oestradiol concentration, ng/L	2822.27 ± 1501.84	2434.00 ± 1523.53	0.853	< 0.001
Serum progesterone concentration, ug/L	0.95 (0.66–1.31)	0.94 (0.64–1.33)	-0.093	0.926
Endometrial thickness, mm	11.25 ± 2.93	11.14 ± 3.08	0.457	0.539
2PN fertilization rate, %	58.73 ± 21.74	58.39 ± 29.06	0.240	0.811
Poly-pronucleus zygote rate, %	0.00 (0.00–0.00)	0.00 (0.00–0.00)	-1.663	0.102
Cleavage rate from 2PN zygote, %	88.52 ± 21.68	86.79 ± 26.74	1.171	0.242
Good-quality embryo rate, %	24.20(1,110/3,581)	28.30(828/2,925)	15.451	< 0.001
No. of available embryo, n	3.64 ± 2.43	3.15 ± 2.32	3.396	0.001
Blastocyst formation rate, %	47.27 ± 33.76	46.77 ± 35.48	0.185	0.853
Cancellation rate, %	31.70 (173/545)	34.10 (186/545)	0.702	0.402

Note: Data are presented as mean ± SD or median (interquartile range) for continuous variables and % (n/N) for categorical variables

The 2PN fertilization rate = number of 2PN zygotes /number of oocytes retrieved; poly-pronucleus zygotes rate = number of poly-pronucleus zygotes/number of oocytes retrieved; cleavage rate from 2PN zygote = number of cleavages from 2PN/number of zygotes; good-quality embryo rate = number of top-quality embryos/number of cleavage-stage embryos from 2PN; percent blastocyst formation = No. of blastocyst formation/ No. of blastocyst culture

*BMI* body mass index, *FSH* Follicle Stimulating Hormone, *Gn* gonadotrophin

Other protocols were minimal stimulation with recombinant follicle-stimulating hormone or human menopausal gonadotrophin, clomiphene citrate,and natural cycle

Comparable 2PN fertilization rate and poly-pronucleus zygote rate were shown in SERa + and SERa- cycles (*P* > 0.05, Table2), but which were higher in SERa + oocytes than in SERa- oocytes (*P* < 0.05, Table 4). No statistical difference in blastocyst formation rate was found in SERa + and SERa- cycles as well as in SERa + and SERa- oocytes (*P* > 0.05, Tables 2 and 4). Good-quality embryo rate was statistically higher in SERa- cycles than in SERa + cycles (*P* > 0.05, Table 2), but the difference was comparable between SERa + and SERa- oocytes (*P* > 0.05, Table 4).

No statistical difference in clinical pregnancy rate, ectopic pregnancy rate, spontaneous abortion rate, live birth rate and premature delivery rate (from 28 to 37 weeks) were found in SERa + and SERa- cycles as well as in SERa + and SERa- oocytes (*P* > 0.05, Tables 3 and 4). The implantation rate was comparable in SERa + and SERa- cycles (Table 3), but it is higher in the group of only SERa- embryo transfer when compared with the group of mixed SERa + and SERa- embryo transfer (*P* < 0.05, Table 4).

**Table 3:** Pregnancy and neonatal outcomes of all patients in SERa + and SERa– cycles

Characteristic	SERa + cycles	SERa—cycles	*χ*^*2*^*/t*	*P-*value^*^
No. of embryo transfer cycles, n	372	359	-	-
No. of embryo transferred,%			0.078	0.780
1	24.73 (92/372)	25.63 (92/359)		
2 or 3	75.27 (280/372)	74.37 (267/359)		
Implantation rate, %	31.67 (210/663)	29.25 (189/645)	0.868	0.352
Clinical pregnancy rate, %	44.89 (167/372)	42.34 (152/359)	0.484	0.487
Singleton pregnancy rate,%	31.50 (117/372)	30.60 (110/359)	0.056	0.813
Twins pregnancy rate,%	12.40 (46/372)	10.90 (39/359)	0.401	0.527
Ectopic pregnancy rate, %	1.10 (4/372)	0.80 (3/359)	0.000	1.000^**^
Live birth rate, %	77.80 (129/167)	79.60 (121/152)	0.111	0.739
Singleton	59.30 ( 99/167)	67.10 (102/152)	2.090	0.148
Twins	18.00 (30/167)	12.50 (19/152)	1.827	0.176
Spontaneous abortion rate, %	17.40 (29/167)	15.80 (24/152)	0.143	0.706
No. of lost follow-up	5	4	-	-
Weeks of gestation, wk	38.28 ± 1.93	38.23 ± 1.72	0.824	0.361
No. of newborns, n	159	140	-	-
Premature delivery rate, %	10.90 (14/129)	15.70 (19/121)	1.282	0.258
Weight of singleton births, kg	3.14 ± 0.60	3.09 ± 0.50	-0.651	0.516
Weight of twin births, kg	2.50 ± 0.45	2.63 ± 0.84	1.036	0.303
Rate of newborn malformation rate, %	1.90 (3/159)	0.70 (1/140)	0.142	0.707^**^

Note: Data are presented as mean ± SD for continuous variables and % (n/N) for categorical variables

Implantation rate = No. of gestational sacs/ No. of transfer embryos, clinical pregnancy rate = No. of pregnancy cycles/No. of embryo transfer cycles, ectopic pregnancy rate = No. of ectopic pregnancy cycles/ No. of embryo transfer cycles, live birth rate = No. of live birth cycles/ No. of pregnancy cycles, spontaneous abortion rate = No. of early and late spontaneous abortion cycles/ No. of clinical pregnancy cycles, premature delivery rate = No. of premature cycles/ No. of live birth cycles, newborn malformation rate = No. of malformed newborns/ No. of live births

Premature delivery is defined as babies born from 28 to 37 weeks

^*^P value calculated with *χ*^*2*^ test or t-test as appropriate

^**^ *P* value calculated with continuity correction *χ*^*2*^ test

**Table 4:** Comparison of embryo development, pregnancy and neonatal outcomes between SERa + and SERa-oocytes from 545 SER + positive cycles (*n* = 6,706)

**Characteristic**	**SERa + oocytes (*n*** **= 1,715)**		**SERa- oocytes (*n* = 4,991)**	P-value^*^
Percentage, %	25.57 (1,715/6,706)		74.43 (4,991/6,706)	-
2PN fertilization rate, %	61.40 (1,053/1,715)		55.90 (2,792/4,991)	< 0.001
Poly-pronucleus zygote rate, %	2.10 (36/1,1715)		0.80 (41/4,991)	< 0.001
Good-quality embryo rate, %	32.30 (311/963)		30.50 (799/2,618)	0.309
Blastocyst formation rate, %	51.30 (241/470)		55.60 (808/1452)	0.098
	**Only SERa + embryo transfer**	**Mixed SERa + and SERa- embryo transfer**	**Only SERa- embryo transfer**	
No. of transferred embryos, n	148	199	316	-
No. of embryo transfer cycles, n	90	97	185	-
No. of lost follow-up	0	3	2	
Implantation rate, %	26.40 (39/148)	23.20 (59/254)	35.40 (112/316)	0.004
Clinical pregnancy rate, %	36.70 (33/90)	43.30 (42/97)	49.70 (92/185)	0.116
EP	1.1(1/90)	1.0(1/97)	1.1 (2/185)	1.000^**^
Live birth rate, %	26.70 (24/90)	32.00 (31/97)	40.50 (75/185)	0.061
Spontaneous abortion rate, %	24.20 (8/33)	16.70 (7/42)	14.10 (13/92)	0.411
Premature delivery rate, %	8.30 (2/24)	25.80 (8/31)	9.30 (7/75)	0.073^**^
No. of newborns, n	30	42	87	
Weeks of gestation, wk	38.14 ± 1.28	37.59 ± 2.11	38.45 ± 1.63	0.912
Weight of singleton births, kg	2.92 ± 0.30	3.06 ± 0.49	3.21 ± 0.71	0.189
Weight of twin births, kg	2.68 ± 0.46	2.39 ± 0.58	2.55 ± 0.24	0.162
Newborn malformation rate, %	3.3 (1/30)	4.80 (2/42)	0.00(0/95)	0.078^**^

Note: Data are presented as mean ± SD for continuous variables and % (n/N) for categorical variables

^*^ *P* value calculated with χ^2^ test or t-test or ANOVA as appropriate

^**^ *P* value calculated with fisher’s exact test

Supplementary tables 1 and 2 have shown the difference in clinical characteristics, embryo development, pregnancy and neonatal outcomes between groups of patients in SERa + cycles with all SERa + oocytes, SERa + cycles with ≥ 50% SERa + oocytes and SERa- cycles. The results indicated the age of the patients in SERa + cycles with all SERa + oocytes or SERa + cycles with ≥ 50% SERa + oocytes were older than in SERa- cycles. The total Gn dose, number of oocytes retrieved, serum estradiol concentration and No. of available embryo were significantly lower in SERa + cycles with all SERa + oocytes and SERa + cycles with ≥ 50% SERa + oocytes than in SERa- cycles. Lower poly-pronucleus zygote rate and higher blastocyst formation rate were found in SERa + cycles with ≥ 50% SERa + oocytes than in SERa- cycles. No obvious difference was found in pregnancy and neonatal outcomes among these groups. One newborn with hydronephrosis was found in SERa + cycles with all SERa + oocytes, but the newborn malformation rate among the three groups were not significant different.

### Neonatal outcomes

There were five SERa + cycles and four SERa- cycles that were lost to follow-up after conception, the rate of loss to follow-up was 0.83%. At last, 159 newborns in SERa + cycles and 140 newborns in SERa- cycles were followed up. Comparable newborn malformation rate was observed between SERa + and SERa- cycles and oocytes (*P* > 0.05, Tables 3 and 4). In SERa + cycles, three newborns had malformations, one with hydronephrosis originating from the group exclusively derived from SERa + embryo transfer, one with persistent left superior vena cava and one with congenital heart disease originating from the group of mixed SERa + and SERa- embryo transfer, the other 156 newborns were healthy babies. In SERa- cycles, one newborn had congenital heart disease, The other 139 newborns were healthy babies.

### Analysis of the risk factors associated with SERa

Univariate analysis and multivariate logistic regression were performed to analyze the factors associated with SERa (Table 5). According to the data shown in Table 1, we incorporated stimulation protocol, oocytes number, the total dose of Gn and serum estradiol concentration into the model. The total dose of Gn and serum estradiol concentration were divided into two groups according to the cutoff value (2157 IU and 1809 ug/L respectively) in following ROC curve analysis. According to logistic regression analysis, oocytes number and total dose of Gn were risk factors for SERa occurrence (aOR = 1.05 and 1.55, *P* < 0.001, Table 5). No significant correlation was found between ovarian stimulation protocol and SERa occurrence (*P* > 0.05, Table 5).

**Table 5:** Univariate analysis and multivariate logistic regression analysis results for risk factors associated with SERa + Cycles

	**Univariate analysis**		**Multivariable analysis**	
	**Crude OR(95%CI)**	***P* value**	**Adjusted OR(95% CI)**	***P* value**
Protocol				
Agonist	1	-	1	-
Antagonist	1.09(0.84–1.43)	0.515	1.23(0.93–1.06)	0.146
Others	0.54(0.37–0.81)	0.002	1.06(0.67–1.70)	0.660
No. of oocytes	1.05(1.03–1.07)	< 0.001	1.05(1.02–1.08)	< 0.001
Total dose of Gn	1.35 (1.06–1.71)	0.015	1.55(1.19–2.02)	0.001
Serum estradiol concentration	1.69(1.32–2.17)	< 0.001	1.16(0.84–1.59)	0.377

**Fig. 2:**
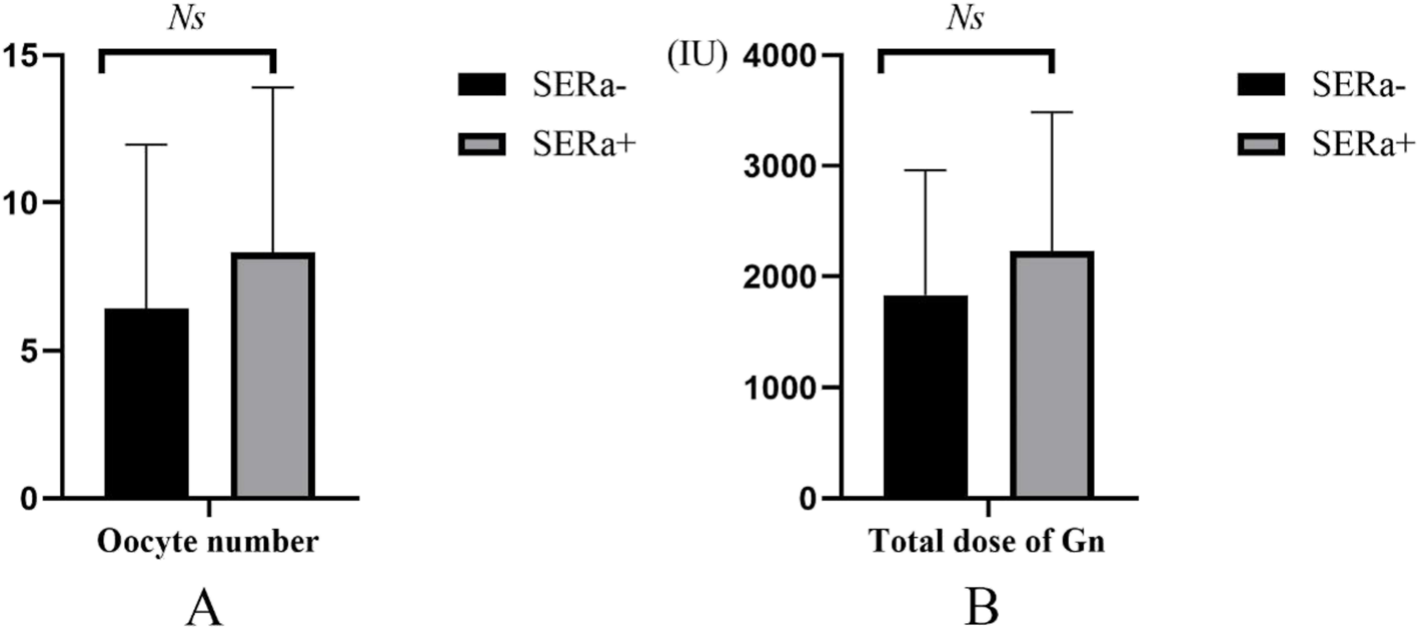
Oocyte number and total dose of Gn for patients with both SERa + and SERa- cycles

According to the logistic regression result, we compared the difference in oocytes number and total dose of Gn in patients with both SERa + and SERa- cycles. 54 patients had both SERa + and SERa- cycles (56 SERa- cycles and 63 SER- cycles). Among these patients, a gradually rising trend of total Gn dose and number of oocytes retrieved were shown in the SERa + cycles than in the SERa- cycles (Fig. 2).

**Fig. 3:**
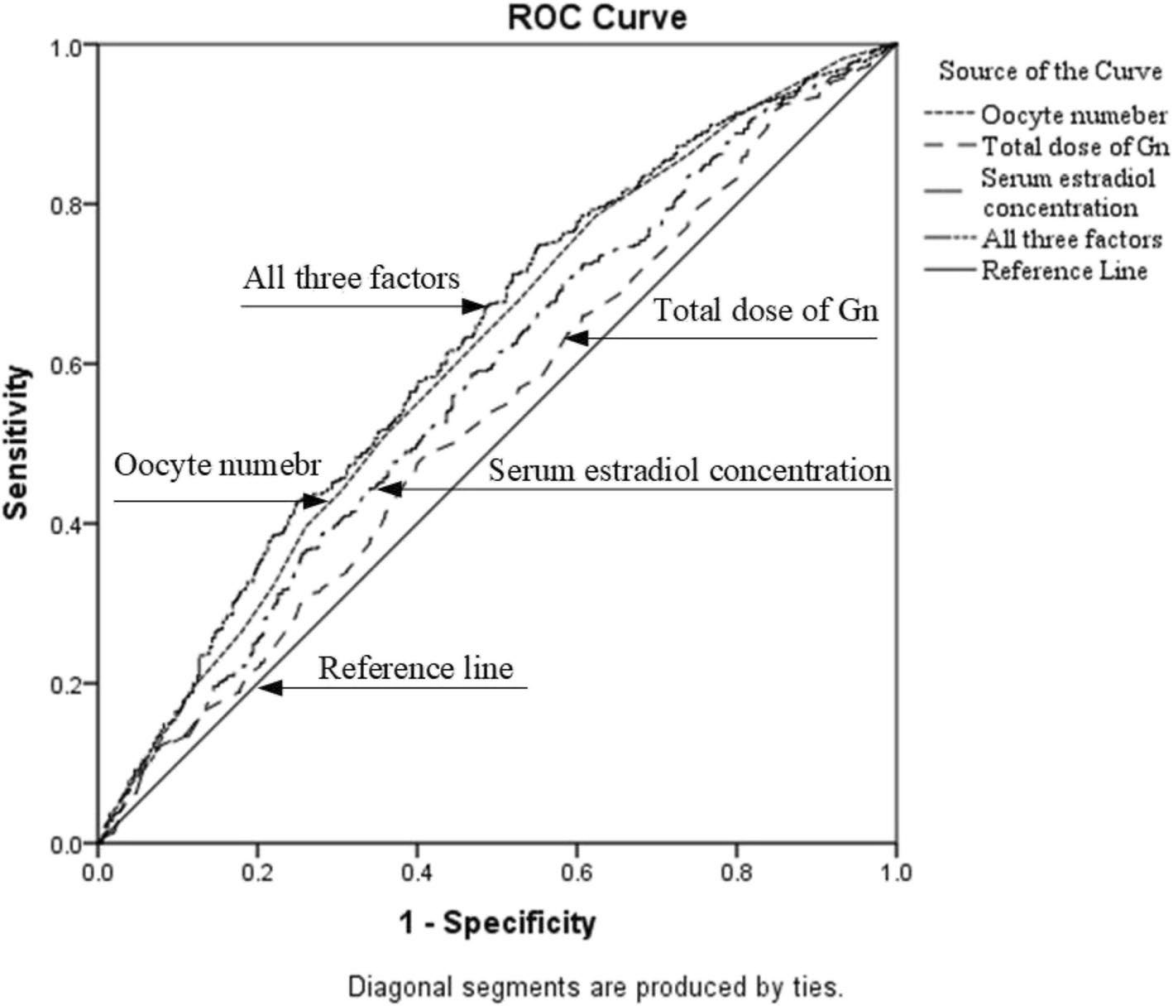
ROC curve analysis of the predictive value of the risk factors for SER + cycles

As shown in Fig. 3, the area under the curve (AUC) of the total dose of Gn, oocytes number and serum estradiol concentration was 0.538 (95% CI 0.504–0.572, *P* = 0.031), 0.608 (95% CI 0.575–0.641, *P* < 0.001) and 0.574 (95% CI 0.540–0.608, *P* < 0.001). The AUC of all three factors was 0.622 (95% CI 0.589–0.655, *P* < 0.001). The cutoff value of the total dose of Gn, serum estradiol concentration and oocyte number for SERa + cycles were 2,157 IU, 1,809 ng/L and 7,which resulted in the largest Youden index, a sensitivity of 48%, 71% and 78%, and a specificity of 59%, 42% and 38%.

## Discussion

This cohort study included 998 patients with 1,090 ICSI cycles, and concretely analyzed the influence of oocytes with SERa on clinical, embryological and newborn outcomes. The rate of loss to follow-up was less than 1%, and propensity score matching was used to minimize possible confounding factors.

Our study showed that the number of retrieved oocytes, the total dose of Gn and the oestradiol level on the day of hCG administration were significantly higher in SERa + cycles than in SERa- cycles, which is similar to the results reported by *Fumiaki Itoi* [15]. In line with this observation, oocyte number and total dose of Gn were correlated with SERa occurrence according to logistic regression analysis. Limited research has been performed on whether the increased oestradiol level is the cause of SERa occurrence. A previous study showed a positive correlation was observed between the presence of SERa and serum oestradiol concentrations on the day of ovulation induction [5], the authors considered high oestradiol levels could be one of the causes of SERa formation. A recent study showed that as aromatase inhibitors did not reduce SERa occurrence, the elevation of oestradiol may not be the cause of SERa occurrence but a consequence [23]. Our study also indicated in the patient with both SERa + and SERa- cycles, an increasing trend of oocytes number and total dose of Gn were shown in SERa + cycles compared with those in SERa- cycles, combined with previous studies’ results, we speculate the increasing of Gn dose may be close with SERa occurrence. However, there is no evidence for the correlation between stimulation protocols and our findings. It may need further study to confirm whether the cycles with more aggressive stimulation have more SERa + oocytes. Besides, our study indicated the age of the patients in SERa + cycles with all SERa + oocytes and SERa + cycles with ≥ 50% SERa + oocytes were older than in SERa- cycles, in line with the result, the total Gn dose, number of oocytes retrieved and serum estradiol concentration were significantly lower in these two groups comparied with in SERa- cycles. Therefore, whether age-related diminished number and quality of oocyte is correlated with SERa occurance deserved to further research. Furthermore, our study once again confirmed the oocyte’s SERa does not obviously affect embryo development and pregnancy outcome, which is similar to some of the previous studies [13, 15], these results suggest that oocytes with SERa should not be discarded and it is relatively safe to injected/inseminated and transferred.

Comparable newborn malformation rate was observed between SERa + and SERa- cycles and no statistical difference in newborn malformation rate was observed among cycles transferred with only SERa + embryos, SERa + and SERa- mixed embryos and SERa- embryos in our study, which is also seen in the previous report [13, 15]. Although some previous studies reported that: genes involved in three main biological processes in SERa + oocytes were significantly down-regulated compared with SERa- oocytes [24]; a higher incidence rate of meiotic cleavage failure during the second polar body extrusion and mitotic cleavage failure after fertilization was present in oocytes with SERa than in oocytes without SERa [9]; SERa reflects intrinsic damage to the oocyte cytoskeleton, namely, alterations in spindle size, chromosome misalignment and cortical actin disorganization [25], our study showed the newborn malformation rate from SERa + cycles was 1.90%, which is below the Chinese national average level (5.6%) [26], whether the newborn malformation occurrence in embryo originated from SERa + oocytes correlated with SERa occurrence was still clear, we consider if no embryo from SERa- oocytes remain, the embryo from SERa + oocytes could be used after informed consent.

The limitation of our study is the neonates were followed to an average age of 24 months, so it is necessary to conduct a longer follow-up and evaluate newborns from SERa + oocytes. Further studies should focus on how to reduce the occurrence of SERa and related mechanisms.

In conclusion, oocyte's SERa is correlated with a number of oocytes retrieved and a higher Gn dose, but it does not affect pregnancy outcomes and increases newborn malformation rate.

## Supplementary Information

**Additional file 1:**

## Data Availability

The data and materials are available from the corresponding author on reasonable requests.

## Abbreviations

SERa: Smooth endoplasmic reticulum aggregates
IVF: In vitro fertilization
ART: Assisted reproductive technology

## References

[1] Sa R (2011). Ultrastructure of tubular smooth endoplasmic reticulum aggregates in human metaphase II oocytes and clinical implications. Fertil Steril.

[2] Machaca K (2004). Increased sensitivity and clustering of elementary Ca2+ release events during oocyte maturation. Dev Biol.

[3] Ozil JP, et al. Egg activation events are regulated by the duration of a sustained [Ca2+]cyt signal in the mouse. Dev Biol. 2005;282(1):39–54. 10.1016/j.ydbio.2005.02.035 15936328

[4] Van Blerkom J. Mitochondrial function in the human oocyte and embryo and their role in developmental competence. Mitochondrion. 2011;11(5):797–813. 10.1016/j.mito.2010.09.012 20933103

[5] Otsuki J (2004). The relationship between pregnancy outcome and smooth endoplasmic reticulum clusters in MII human oocytes. Hum Reprod.

[6] Ebner T (2008). Prognosis of oocytes showing aggregation of smooth endoplasmic reticulum. Reprod Biomed Online.

[7] Akarsu C (2009). Smooth endoplasmic reticulum aggregations in all retrieved oocytes causing recurrent multiple anomalies: case report. Fertil Steril.

[8] The Istanbul consensus workshop on embryo assessment (2011). proceedings of an expert meeting. Hum Reprod.

[9] Otsuki J (2018). A higher incidence of cleavage failure in oocytes containing smooth endoplasmic reticulum clusters. J Assist Reprod Genet.

[10] Hattori H (2014). Deliveries of babies with normal health derived from oocytes with smooth endoplasmic reticulum clusters. J Assist Reprod Genet.

[11] Setti AS (2016). Oocytes with smooth endoplasmic reticulum clusters originate blastocysts with impaired implantation potential. Fertil Steril.

[12] Braga DP (2013). Influence of oocyte dysmorphisms on blastocyst formation and quality. Fertil Steril.

[13] Shaw-Jackson C (2016). Oocytes affected by smooth endoplasmic reticulum aggregates: to discard or not to discard?. Arch Gynecol Obstet.

[14] Mateizel I (2013). Deliveries of normal healthy babies from embryos originating from oocytes showing the presence of smooth endoplasmic reticulum aggregates. Hum Reprod.

[15] Itoi F (2017). Clinical outcomes after IVF or ICSI using human blastocysts derived from oocytes containing aggregates of smooth endoplasmic reticulum. Reprod Biomed Online.

[16] Itoi F (2016). Embryological outcomes in cycles with human oocytes containing large tubular smooth endoplasmic reticulum clusters after conventional in vitro fertilization. Gynecol Endocrinol.

[17] Restelli L (2015). The impact of Alpha/ESHRE consensus regarding oocytes with aggregates of smooth endoplasmic reticulum (SERa) on in vitro fertilization outcome. J Assist Reprod Genet.

[18] The Vienna consensus (2017). report of an expert meeting on the development of ART laboratory performance indicators. Reprod Biomed Online.

[19] Ferreux L (2019). Is it time to reconsider how to manage oocytes affected by smooth endoplasmic reticulum aggregates?. Hum Reprod.

[20] Massarotti C (2021). Occurrence of smooth endoplasmic reticulum aggregates in metaphase II oocytes: relationship with stimulation protocols and outcome of ICSI and IVF cycles. Hum Reprod.

[21] Li Y (2019). Investigating the impact of local inflammation on granulosa cells and follicular development in women with ovarian endometriosis. Fertil Steril.

[22] Fang T (2015). Predictive value of age-specific FSH levels for IVF-ET outcome in women with normal ovarian function. Reprod Biol Endocrinol.

[23] Saito H (2019). A higher incidence of smooth endoplasmic reticulum clusters with aromatase inhibitors. Reprod Med Biol.

[24] Stigliani S (2018). Presence of aggregates of smooth endoplasmic reticulum in MII oocytes affects oocyte competence: molecular-based evidence. Mol Hum Reprod.

[25] Dal Canto M (2017). Dysmorphic patterns are associated with cytoskeletal alterations in human oocytes. Hum Reprod.

[26] Yu M (2015). The survey of birth defects rate based on birth registration system. Chin Med J (Engl).

