# Supplementary material for: The impact of oocytes containing smooth endoplasmic reticulum aggregates on assisted reproductive outcomes: a cohort study
Source: BMC Pregnancy Childbirth. 2022 Nov 14;22:838. doi: 10.1186/s12884-022-05141-9 (PMC9664725; doi:10.1186/s12884-022-05141-9)
Supplement: Supplementary file 1 — Additional file 1: [file 12884_2022_5141_MOESM1_ESM.docx]

**Supplementary Table** **1 Clinical characteristics and embryo development** **for**

**patients in** **SERa+ cycles with all SERa+ oocytes, SERa+ cycles with ≥50% SERa+ oocytes and SERa– cycles.**

| **Characteristic** | **SERa+ cycles with all SERa+ oocytes**  **（n=24）** | **SERa+ cycles with**  **≥50% SERa+ oocytes (n=127)** | **SERa- cycles**  **(n=545)** |
| --- | --- | --- | --- |
| Age | 38.29±5.08 ^a^ | 35.47±5.25 ^b^ | 33.97±5.63 ^c^ |
| Protocol, % |  |  |  |
| Agonist | 33.30 (8/24) ^a^ | 53.54 (68/127) | 60.60(330/545) ^c^ |
| Antagonist | 12.50 (3/24) | 26.00(33/127) | 31.00 (169/545) |
| Others | 54.17 (13/24) ^a^ | 20.47 (26/127) ^b^ | 8.40 (46/545) ^c^ |
| Total dose of Gn, IU | 1547.92±1213.64 ^a^ | 2206.87±1126.83 | 2071.28±880.37 ^c^ |
| Initial dose of Gn, IU | 176.04±88.31 | 199.41±70.60 | 188.96±63.86 |
| Duration of Gn, days | 7.25±3.89 ^a^ | 10.14±3.36 | 10.35±2.94^c^ |
| No. of oocytes  retrieved, n | 3.08±2.84 ^a^ | 7.90±5.61^b^ | 9.89±7.14 ^c^ |
| No. of SERa- oocytes, n | - | 2.66±2.50 ^b^ | 9.89±7.14 ^c^ |
| Serum oestradiol concentration, ng/L | 527.50  (298.00-970.25) ^a^ | 1582.00  (926.00-3169.00) ^b^ | 2158.00  (1186.50-3609.00) ^c^ |
| Serum progesterone concentration, ug/L | 0.87 (0.44-1.26) | 1.03(0.68-1.33) | 0.94 (0.64-1.33) |
| Endometrial thickness, mm | 10.41±2.63 | 10.93±3.16 | 11.14±3.08 |
| 2PN fertilization rate, % | 74.30 (55/74) ^a^ | 60.60 (157/259) | 56.60 (3049/5388) ^c^ |
| Poly-pronucleus zygote rate, % | 2.70 (2/74) | 7.30 (19/259) ^b^ | 0.80 (44/5388) ^c^ |
| Cleavage rate from 2PN zygote, % | 90.21±23.80 | 88.37±22.47 | 86.79±26.74 |
| Good-quality embryo rate, % | 30.20 (16/53) | 32.00 (186/581) | 28.30 (828/2,925) |
| No. of available embryo, n | 1.75±1.33^a^ | 2.64±2.01^b^ | 3.15±2.32^c^ |
| Blastocyst formation rate, % | 42.90 (3/7) | 39.20 (100/255) ^b^ | 51.30 (690/1345) ^c^ |
| Cancellation rate, % | 37.50 (9/24) | 31.50 (40/127) | 34.10 (186/545) |

**Supplementary table 2** **Pregnancy and neonatal outcomes of all patients**

**in SERa+ cycles with all SERa+ oocytes, SERa+ cycles with ≥50% SERa+ oocytes and SERa– cycles.**

| **Characteristic** | **SERa+ cycles with all SERa+ oocytes** | **SERa+ cycles with ≥50% SERa+ oocytes** | **SERa - cycles** |
| --- | --- | --- | --- |
| No. of embryo transfer cycles, n | 15 | 87 | 359 |
| No. of embryo transferred, % |  |  |  |
| 1 | 33.33 (5/15) | 23.00 (20/87) | 25.63 (92/359) |
| 2 or 3 | 66.67 (10/15) | 77.00 (67/87) | 74.37 (267/359) |
| Implantation rate, % | 28.00 (7/25) | 25.80 (40/155) | 29.25 (189/645) |
| Clinical pregnancy rate, % | 33.33 (5/15) | 35.63 (31/87) | 42.34 (152/359) |
| Singleton pregnancy rate, % | 13.33 (2/15) | 22.30 (20/87) | 30.60 (110/359) |
| Twin pregnancy rate, % | 20.00 (3/15) | 11.49 (10/87) | 10.90 (39/359) |
| Ectopic pregnancy rate, % | 0 | 1.15 (1/87) | 0.80 (3/359) |
| Live birth rate, % | 80.00 (4/5) | 83.90 (26/31) | 79.60 (121/152) |
| Singleton | 40.00 (2/5) | 54.84 (17/31) | 67.10 (102/152) |
| Twins | 40.00 (2/5) | 29.03 (9/31)^b^ | 12.50 (19/152)^c^ |
| Spontaneous abortion rate, % | 20.00 (1/5) | 9.68 (3/31) | 15.80 (24/152) |
| No. of lost follow-up | 0 | 1 | 4 |
| Weeks of gestation, wk | 38.32±1.25 | 38.14±1.49 | 38.23±1.72 |
| No. of newborns, n | 6 | 35 | 140 |
| Premature delivery rate, % | 0 | 11.54 (3/26) | 15.70 (19/121) |
| Weight of singleton births, kg | 3.15±0.14 | 3.05±0.32 | 3.09±0.50 |
| Weight of twin births, kg | 2.48±0.57 | 2.66±0.60 | 2.63±0.84 |
| Rate of newborn malformation rate, % | 16.70 (1/6) | 2.86 (1/35) | 0.70 (1/140) |
